# Proteome dataset of *Hemileia vastatrix* by LC–MS/MS label-free identification

**DOI:** 10.1016/j.dib.2022.108433

**Published:** 2022-07-03

**Authors:** Natália Faustino Cury, Daiane Gonzaga Ribeiro, Jonathan Dias de Lima, Pollyana da Nóbrega Mendes, Diana Fernandez, Wagner Fontes, Mariana S. Castro, Marcelo V. Sousa, Natália F. Martins, Angela Mehta

**Affiliations:** a Embrapa Recursos Genéticos e Biotecnologia, Parque Estação Biológica, PqEB, Av. W5 Norte (final), Brasília, DF 70770-917, Brazil; b Centro de Análises Proteômicas e Bioquímicas, Programa de Pós-Graduação em Ciências Genômicas e Biotecnologia, Universidade Católica de Brasília, Taguatinga, DF 71966-700, Brazil; c Laboratório de Inorgânica e Materiais (LIMA - IQ/UnB), Departamento de Ciências de Materiais, Universidade de Brasilia, Planaltina, DF 73.345-010, Brazil; d IRD - Institut de Recherche pour le Développement, PHIM Plant Health Institute, Univ Montpellier, IRD, CIRAD, INRAE, Institut Agro, Montpellier, France; e Laboratório de Bioquímica e Química de Proteínas, Departamento de Biologia Celular, Instituto de Ciências Biológicas, Universidade de Brasilia, Brasilia, DF, 70919-900, Brazil; f Embrapa Agroindustria Tropical, Rua Dra. Sara Mesquita, no 2.270, Bairro Planalto do Pici, Fortaleza, CE 60511-110, Brazil

**Keywords:** *Hemileia vastatrix*, Coffee rust, Proteome, LC–MS/MS

## Abstract

Here we describe the proteome of the fungus *Hemileia vastatrix* by label free mass spectrometry (LC–MS/MS). *H. vastatrix* is the causal agent of coffee rust disease, causing great economic losses in this crop. The objective of our work was to identify *H. vastatrix* proteins potentially involved in host colonization and infection, by exploring the shotgun proteomics approach. A total of 742 proteins were identified and are associated with several crucial molecular functions, biological processes, and cellular components. The proteins identified contribute to a better understanding of the metabolism of the fungus and may help identify target proteins for the development of specific drugs in order to control coffee rust disease. All data can be accessed at the Centre for Computational Mass Spectrometry – MassIVE MSV000087665 -https://massive.ucsd.edu/ProteoSAFe/dataset.jsp?task=cc71ad75f767451abe72dd1ce0019387

## Specifications Table

**Table:** 

Subject	Biological sciences
Specific subject area	Omics: Proteomics Fungus proteomics
Type of data	Raw data by nano UPLC-MS/MS
How the data were acquired	• LC–MS/MS was performed on an LTQ Orbitrap Elite mass spectrometer (Thermo Fisher Scientific) coupled to an Ultimate 3000 RSLCnano UPLC system (Thermo Scientific Dionex). • MS was operated in DDA mode, acquiring precursor ions at 120000 resolution and fragmenting the top 15 precursors by HCD. • MS/MS data was analysed by de novo sequencing and sequence database searching.
Data format	The set of spectra obtained was stored in .RAW.
Description of data collection	Urediniospores of *H. vastatrix* were collected from infected *Coffea arabica* L. plants and spores with a germination rate higher than 80% were used for germination.
Data source location	• Institution: Embrapa Recursos Genéticos e Biotecnologia • City/Town/Region: Brasília • Country: Brazil
Data accessibility	Public**Repository name:** Centre for Computational Mass Spectrometry – MASSIVE**Data identification number:** MassIVE MSV000087665 **Direct URL to data:**https://massive.ucsd.edu/ProteoSAFe/dataset.jsp?task=cc71ad75f767451abe72dd1ce0019387**Instructions for accessing these data:**Web access: https://massive.ucsd.edu/MassIVE ID = MSV000087665PorteomeXchange ID = PXD026810DOI = 10.25345/C5FK0D

## Value of the Data

• The proteins identified in this study contribute to better understand the metabolism of *Hemilea vastatrix*.
• The data obtained can help researchers and agricultural industries to identify target proteins for the development of specific drugs in order to control coffee rust disease.
• The dataset of *H. vastatrix* proteins represents valuable information that contributes to the Pucciniales proteome repertoire.

## 1 Data Description

**Fig. 1:**
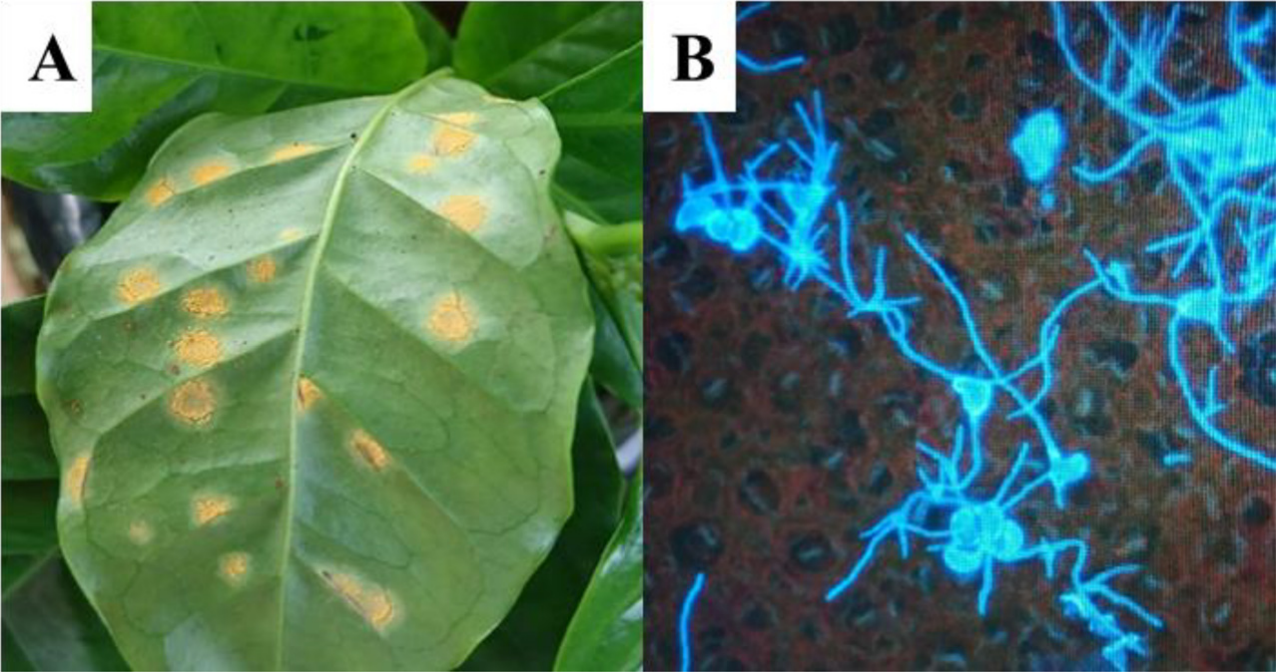
(A) *Hemileia vastatrix* urediniospores germinated at coffee (*Coffea* ar*abica*) leaf surface. (B) Rust (*H. vastatrix*) pustules on a coffee leaf.

**Fig. 2:**
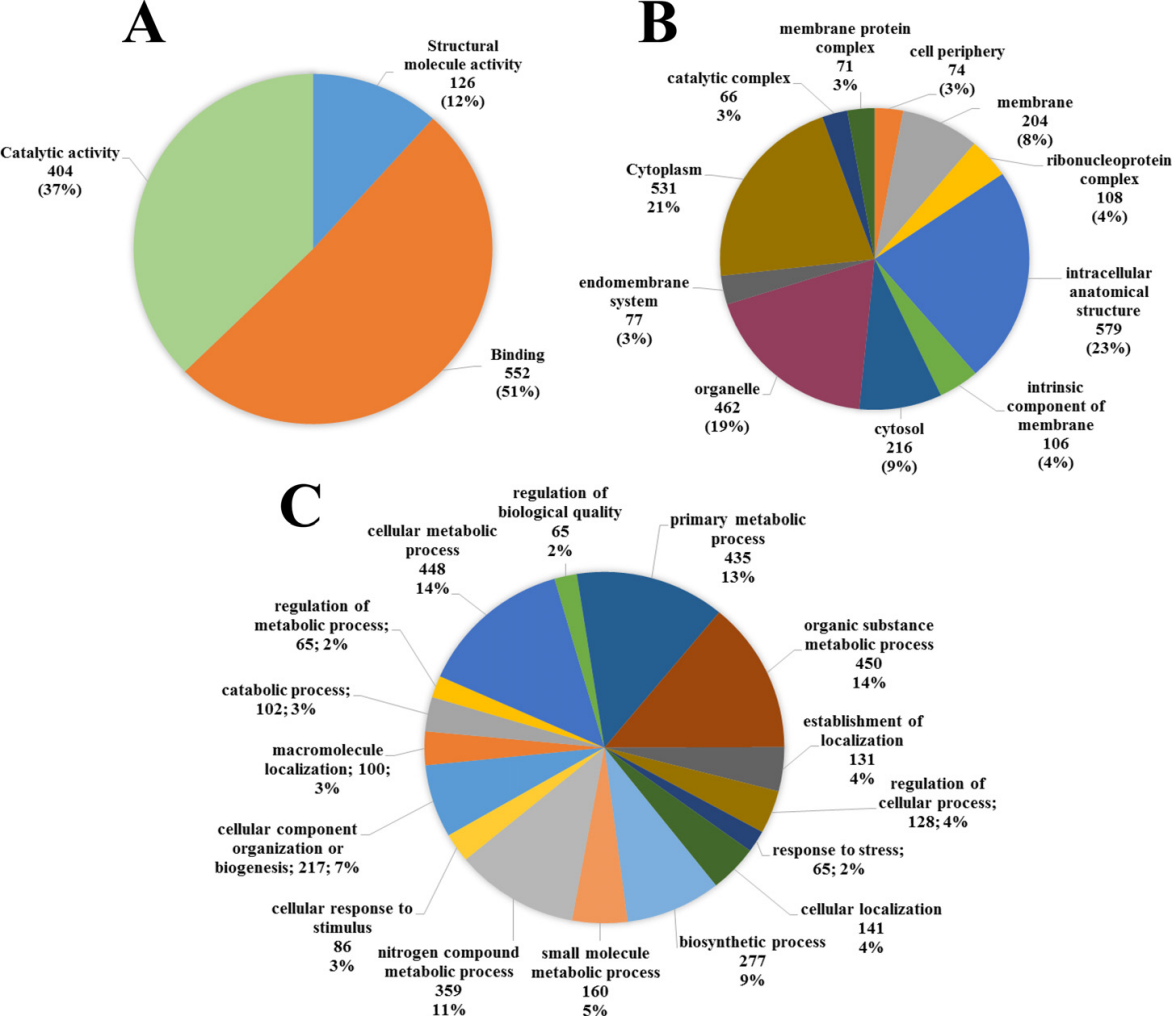
Diagram showing the molecular functions (A), biological processes (B), and cellular sites (C) of identified proteins in germinating urediniospores of *Hemileia vastatrix.*

The dataset described here was obtained from the proteome analysis of the fungus *Hemileia vastatrix* Berkeley and Broome (Basidiomycota, Pucciniales). A total of 742 proteins of *H. vastatrix* were identified using the PEAKS software and the proteins were deposited in the MassIVE repository under the ID MSV000087665. All the files presented in the MassIVE repository are described in Table 1. The proteins identified in *H. vastatrix* germinating urediniospores (Fig. 1) were classified according to their molecular functions, cellular components, and biological processes categories (Fig. 2).

**Table 1:** Description of the folders deposited in the Massive / ProteomeXchange repositories.

Files	File description
Methods_and_protocols	Configuration used in chromatography and mass spectrometry analyses.
Raw_spec_files	Set of obtained spectra.
Sequence_database	Database sequences retrieved from the Uniprot repository in May 2021, filtered for the order Pucciniales, taxon ID 5258.
Peaklist_files	File in the MGF (Mascot generic file) format, containing the peaklist used for identification in the Peaks software, required for validation in the MassIVE workflow.
Result_files	File in the MZID (mzIdentML) format, containing the identification results exported by the Peaks software, based on the search of the peaklist in the database, both mentioned above, also used for validation in the MassIVE workflow.
Search_engine_files	All files generated by Peaks software, in which the complete set of spectra and protein identification were analyzed. The tables of identified peptides and proteins can be found in the "export" subfolder.
Supplementary_files	The iteractive sequence of mappings (Uniprot, DB2DB on BioDBnet and BLASTKoala) and their results.

## 2 Experimental Design, Materials and Methods

The urediniospores of *H. vastatrix* (race II, isolate Hv01) were collected from artificially infected leaves of *C. arabica* (var. Catuaí Amarelo) plants grown in a greenhouse (Fig. 1). Approximately 10 mg of urediniospores were spread in 10 mL distilled water and allowed to germinate in Petri dishes kept in the dark at 24 °C.

Germinated spores (> 80%) and non-germinated spores (altogether called germinating urediniospores (gU) sample) were harvested after 24 hours by centrifugation at 12000 rpm for 2 min. The gU from five Petri dishes were collected into one single tube to form the *H. vastatrix* gU sample used for protein extraction as described by Ribeiro et al. [1]. For tryptic digestion, the sample was solubilized with 60 µL of 50 mM ammonium bicarbonate (NH_4_HCO_3_ pH 8.5), then 25 µL of RapiGestTM SF – Waters (0.2% v/v) was added. The sample was reduced with dithiothreitol (100 mM), alkylated with iodoacetamide (300 mM) and proteins were digested using 200 ng of trypsin at 37 °C for 19 h.

The peptides were desalted as described in Rappsilber et al. [2], with some modifications [1], solubilized with 0.1% formic acid and injected into an Ultimate 3000 RSLCnano UPLC (Thermo Scientific Dionex) for reversed-phase nano-chromatography. A total of 1 µg of solubilized peptides were injected into a trap column (2 cm × 100 µm), containing ReproSil-Pur C18-AQ 5 µm reverse phase resin (Dr. Maisch GmbH, Germany) in triplicate. The sample was eluted from the trap column to the analytical column (32 cm × 75 µm containing ReproSil-Pur C18-AQ 3 µm reverse phase resin -Dr. Maisch GmbH, Germany). A gradient of 2–35% acetonitrile, 0.1% formic acid was used and the sample was sprayed into the ionization source of the spectrometer.

The LTQ Orbitrap Elite mass spectrometer was operated in data-dependent acquisition (DDA) mode, generating MS1 spectra in the Orbitrap analyzer (with resolution of 120 000 FWHM at 400 m/z) between the masses 300–1650 m/z and dynamic exclusion of 10 ppm. The 15 most intense ions were chosen for each MS1 spectrum automatically with charges higher than two and directed toward higher energy collision-induced dissociation (HCD). The configuration for HCD was: 2.0 m/z isolation window with automatic gain control (AGC) of 1 × 10^6^, and maximum fill time of 100 ms, with normalized collision energy at 35% and threshold for the selection of 3000.

Alignment of spectra and quantification of peptides were performed using Progenesis® QI for proteomics v.1.0 software [3] and proteins were identified using Peaks® 7.0 software [4]. The sequences were deduced from the fragmentation information and the search performed in the Uniprot (Universal Protein Resource) repository in May 2021, filtered to the order Pucciniales (Taxon ID: 5258). The search was performed based on de novo sequencing and PSM with the following parameters: tolerance for the mass of the precursor of 10 ppm, and of 0.05 Da for the fragments, tolerance of up to 2 missing cleavages, carbamidomethylation of cysteines as a fixed modification and methionine oxidation as a variable modification. Protein identifications were considered as being reliable at FDR *<* 1%, presenting at least two unique peptides. Finally, the proteins identified were functionally annotated using Blast2GO software [5].

## Ethics Statements

This research involved neither human participants nor animals.

## CRediT authorship contribution statement

**Natália Faustino Cury:** Writing – original draft, Formal analysis. **Daiane Gonzaga Ribeiro:** Methodology, Writing – original draft. **Jonathan Dias de Lima:** Methodology. **Pollyana da Nóbrega Mendes:** Methodology, Writing – original draft. **Diana Fernandez:** Methodology, Investigation. **Wagner Fontes:** Formal analysis, Data curation, Software. **Mariana S. Castro:** Software. **Marcelo V. Sousa:** Resources. **Natália F. Martins:** Project administration, Resources. **Angela Mehta:** Investigation, Supervision, Writing – review & editing.

## Declaration of Competing Interest

The authors declare that they have no known financial interests and personal relationships that could inappropriately influence the work reported in this paper.

## Data Availability

MassIVE MSV000087665 (Original data) (Centre for Computational Mass Spectrometry – MASSIVE).

## References

[1] Ribeiro D.G., de Almeida R.F., Fontes W., de Souza Castro M., de Sousa M.V., Ricart C.A.O., da Cunha R.N.V., Lopes R., Scherwinski-Pereira J.E., Mehta A. (2019). Stress and cell cycle regulation during somatic embryogenesis plays a key role in oil palm callus development. J. Proteom..

[2] Rappsilber J., Mann M., Ishihama Y. (2007). Protocol for micro-purification, enrichment, pre-fractionation and storage of peptides for proteomics using StageTips. Nat. Protoc..

[3] Välikangas T., Suomi T., Elo L.L. (2018). A comprehensive evaluation of popular proteomics software workflows for label-free proteome quantification and imputation. Briefings Bioinf..

[4] Ma B., Zhang K., Hendrie C., Liang C., Li M., Doherty-Kirby A., Lajoie G. (2003). PEAKS: powerful software for peptide de novo sequencing by tandem mass spectrometry. Rapid Commun. Mass Spectrom..

[5] Conesa A., Götz S. (2008). Blast2GO: a comprehensive suite for functional analysis in plant genomics. Int. J. Plant Genom..

